# Reliability assessment of the malay version visual comfort questionnaire for schoolchildren with visual impairment

**DOI:** 10.1371/journal.pone.0333228

**Published:** 2025-10-07

**Authors:** Pui Theng Yong, Zainora Mohammed, Norliza Mohamad Fadzil, Mohd Harimi Abd Rahman, Mohd Izzuddin Hairol, Sumithira Narayanasamy

**Affiliations:** 1 Centre for Rehabilitation and Special Needs Study, Faculty of Health Sciences, Universiti Kebangsaan Malaysia, Kuala Lumpur Malaysia; 2 Centre for Community Health Studies, Faculty of Health Sciences, Universiti Kebangsaan Malaysia, Kuala Lumpur Malaysia; ISSEP Kef: Universite de Jendouba Institut Superieur du Sport et de l'Education Physique du Kef, TUNISIA

## Abstract

Lighting adjustments are crucial in vision rehabilitation to enhance visual function and promote visual comfort. However, there is a lack of validated questionnaires to assess lighting needs among schoolchildren with visual impairment. Given the limited knowledge of their specific need for reading and learning activities, a translated and reliable instrument is essential for guiding evidence-based support. This study aims to translate, adapt, and assess face validity, content validity and reliability of the Malay version Visual Comfort Questionnaire (VCQ-M). This is a cross-sectional study that was carried out from November 2022 to January 2023. Forward-backwards translation was used to translate the items in the questionnaire from English into Malay. Face and content validity of the translated questionnaire were determined to ensure comprehensibility and cultural adaptability. Ten normal-sighted adults participated in face validation. Five optometrists were recruited as expert panels to rate the questionnaire items. The inter-rater reliability of the questionnaire was assessed using Fleiss’ Kappa and the inter-class correlation coefficient (ICC). To determine the internal consistency of VCQ-M, 42 subjects with visual impairment from a special secondary school were recruited in this study to complete the VCQ-M after reading a paragraph comprised of 50 words under lighting of 125 lux. Internal consistency of VCQ-M was analysed using Cronbach’s alpha with composite reliability for confirmation. The face validity showed 91.0% of overall agreement, while the content validity index for the item was above 0.80 at item-level and scale-level. The translated VCQ-M has acceptable Fleiss’ Kappa inter-rater reliability for relevance (κ = 0.54) and comprehensibility (κ = 0.44), as well as ICC inter-rater reliability for relevance (ICC(3, 5) = 0.88) and comprehensibility (ICC(3, 5) = 0.73). The Cronbach’s alpha (α = 0.92) showed acceptable internal consistency and confirmed with Dijkstra-Henseler’s rho (ρA = 0.95) and Jöreskog’s rho (ρc = 0.94). The VCQ-M shows an acceptable face validity index, content validity index, inter-rater reliability and internal consistency. This study represents a first-stage validation focused on internal consistency. Construct validity was not assessed, and thus the psychometric evaluation is partial and preliminary. However, the findings support using VCQ-M as a reliable and valid questionnaire to assess visual comfort among Malay-speaking schoolchildren with visual impairment.

## Introduction

Lighting adjustment and modification are important in vision rehabilitation, particularly for schoolchildren with visual impairments. Tailored lighting conditions can enhance residual vision, reduce visual fatigue, and improve the ability to access educational materials and participate effectively in classroom activities [1–2]. Modifying the lighting level for reading tasks is commonly recommended by optometrists to enhance visual functions and promote visual comfort, as lighting requirement vary depending on the condition and cause of vision loss [3]. However, lighting modifications are typically recommended in private home settings or workplace and are less commonly applied in classrooms. A study by McIntosh et al. (2023) found that visually impaired patients who received personalised task lighting recommendations experienced significant improvements in reading speed and comfort in home settings [4].

Visual comfort describes how individuals perceive and respond to the visual aspects of surroundings [5], which may influence individuals’ behaviour, efficiency, and well-being within a space [6]. Architects often conduct a visual comfort assessment during the lighting design of a building using questionnaires [7]. However, most of these survey questionnaires were developed for the general population, and their validity may not be determined. In addition, ophthalmologists and optometrists often rely on qualitative approaches when measuring visual comfort among their patients [8–9]. While this method allows tailored lighting requirements to be prescribed based on an individual’s visual functions and subjective feedback, it may not be effective for quantifying improvements following intervention. Moreover, the relationship between visual function and visual comfort has not been thoroughly investigated, and the changes of lighting needs due to ocular conditions might not be readily apparent.

Lighting needs for optimal visual performance and visual comfort among adults with visual impairment and schoolchildren with normal vision have been investigated in the past [10,11]. For instance, a home-used lighting assessment for older adults with visual impairment has been developed and validated by Şahlı & İdil (2019) [10]. Similarly, a recent study has validated a questionnaire to measure the perception of visual comfort among schoolchildren with normal vision from a mainstream school [11]. To the best of our knowledge, no published study validates a visual comfort questionnaire specifically for schoolchildren with visual impairment. Furthermore, the relationship between visual functions and visual comfort among schoolchildren with vision impairment has not been previously investigated. As a result, changes in lighting needs due to ocular conditions may not be well understood.

Investigating the optimal lighting level can be challenging because schoolchildren with visual impairment often have different demands [12–13]. Furthermore, they may have different levels of sensitivity to lighting depending on the type of ocular disease and its severity [3,14]. For instance, photophobia was a common complaint among patients with ocular diseases such as cataract, nystagmus, glaucoma, and macular degeneration [14–17]. In contrast, many patients also reported reduced vision under dim lighting conditions [14–17]. Previous studies have also shown that higher illumination levels can improve the visual functions of individuals with visual impairment but it may compromise visual comfort [8,15,18].

Recommendation and modification of physical environment and lighting level in special schools for visually impaired and blind children were often discussed and emphasized by building environment engineers and designers, and occupational optometrists, to provide a conducive learning environment for them [19–21]. However, most of previous studies on the physical environment, lighting levels, and their effect on visual comfort were qualitative and relied entirely on the subjective perception of subjects [15,22,23]. Consequently, there is a lack of statistical data quantifying the relationship between lighting level and visual comfort. Given the potential interaction between visual function and visual comfort, both factors should be measured to determine optimal lighting conditions. One qualitative method for measuring visual comfort that has been reported involved survey questionnaires that assess visual annoyance and comfort, as well as visual stimulation factors and mood [24]. However, the questionnaire is unavailable in Malay, the national language and medium of instruction in Malaysian national schools [25–27]. Therefore, to use the questionnaire in the Malaysian context, it is essential to undertake a rigorous translation, adaptation and validation process to ensure that the Malay version is as similar as possible to the original [25,27]. Additionally, validating and testing the reliability of the translated questionnaire is crucial to maintain its quality and integrity, especially when administered to the intended respondents [28].

Given the lack of standardised lighting guidelines for reading and learning activities in Malaysia, developing a translated, culturally adapted, and psychometrically validated quantitative questionnaire is essential. Adequate lighting is a critical factor influencing visual function, and optimal visual function is fundamental to effective learning. Therefore, identifying appropriate lighting levels that improve visual comfort is necessary to support academic performance and overall well-being among schoolchildren with visual impairment. To ensure the reliability and relevance of the questionnaire, it must be linguistically adapted to meet the comprehension levels of schoolchildren with visual impairment. Therefore, this study aimed to translate and adapt the Visual Comfort Questionnaire to the Malay language and to assess its validity and reliability in schoolchildren with visual impairment.

## Materials and methods

### Ethical approval

This study protocol has been approved by the Ethics and Research Committee of Universiti Kebangsaan Malaysia (UKM PPI/111/8/JEP-2022–560). At the same time, written approval was obtained from the Ministry of Education (KPM.600–3/2/3-eras(12821)), Special Education Division of Malaysia (KPM.600–2/1/4 JLD.6 (81)) and school authorities. Written informed consent was obtained from the participants and their parent/ guardian through the school administrative staff.

### Study design

A cross-sectional survey study was conducted to determine the validity and reliability of the Malay version Visual Comfort Questionnaire. Participants’ recruitment and data collection were conducted from 09/11/2022 to 25/02/2023.

### Visual comfort questionnaire

The questionnaire’s translation, adaptation and validation are necessary as the original version was English and not specifically designed for visually impaired schoolchildren. These steps were undertaken to ensure the questionnaire could be appropriately implemented in a study investigating visual functions and visual comfort under different lighting levels among schoolchildren with visual impairment. To identify the most suitable questionnaire for this purpose, a literature search was conducted from September 2022 to November 2022 to find published articles that used questionnaires to assess visual comfort. The search terms include ‘questionnaire’, ‘visual comfort’, ‘lighting’, ‘illumination level’, and ‘reading’. Articles were filtered based on the following criteria: 1) visual comfort was quantitatively measured using a questionnaire, 2) participants were asked to rate their responses in relation to the lighting level during reading tasks, and 3) the questionnaire items focused specifically on task lighting without considering other factors like day lighting, type of artificial light source, or light temperature. The search yielded 320 articles, of which seven met the first criterion [24,29–34]. Further filtering based on the remaining two criteria resulted in a single article by Lee et al. (2014) [24]. Table 1 summarises the questionnaire selection during literature search. Key components present or lacking in the identified questionnaires from previous studies are shown. The questionnaire developed by Lee et al. (2014) addressed the quantitative measure of visual comfort and lighting perception in reading tasks and focused explicitly on task lighting [24], which makes it the most appropriate instrument for adaptation in this study. A structured comparison is provided to justify this selection. The selected questionnaire used by Lee et al. (2014) consisted of three domains: visual annoyance and comfort (six items), visual perceptions of stimulation factors (six items), and mood (seven items). The questionnaire utilised a seven-point rating scale [24].

**Table 1:** Shortlisted questionnaire based on the set criteria.

Author (Year)	Questionnaire	Domain	Targeted Users	Validation	Criteria Met		
					Quantitative measure	Lighting perception in reading	Specific focus on task lighting
Avcı & Memikoğlu (2017) [29]	Adopted from Office Lighting Survey (Eklund & Boyce 1996)	None	University students (19–30 years old)	N	Y	Y	N
Chiou et al. (2020) [30]	Visual Comfort Questionnaire	• Physiological symptoms • Visual annoyance & tasks performance • User preference • General conclusion	University students (Average: 19 years old)	N	Y	N	N
Erell et al. (2014) [31]	Unnamed	• Room assessment & quality of visual environment • Preference in setting • Perception of indoor climate	17–48 years old	N	Y	Y	N
Lee et al. (2014) [24]	Survey questionnaires for visual annoyance and comfort Survey questionnaires for visual stimulation factors and mood	• Visual annoyance and comfort • Visual perception of stimulation factors • Mood	University students	N	Y	Y	Y
Liu et al. (2023) [32]	Lighting perceptions questionnaire	• Daylight sufficiency • Glare • Daylight satisfaction	Middle school children	N	Y	N	N
Magero et al. (2023) [33]	Developed based on parameters in SBTool (Sustainable Building Tool)	• Physiological symptoms • Perception of brightness • Task performance	Secondary school children	N	Y	Y	N
Yunitsyna & Toska (2023) [34]	Daylight satisfaction survey	• Lighting satisfaction • Glare intensity • Shading devices used • Artificial lighting usage • Daylight control strategies	Teachers & students in an art school	N	Y	N	N

Y = Yes; N = No.

### Translation

The translation and cross-cultural adaptation process in this study followed the methodology recommended by the World Health Organization (2009) [35], which is widely applied by other authors for instrument translation and adaptation [36–38]. After permission was granted from the authors of the original survey questionnaire, 19 items from both parts of the original questionnaires were compiled into a single questionnaire. The questionnaire was given to two independent translators for forward translation. Both translators were native Malay speakers; one is a qualified optometrist, and the other is a certified English-Malay translator. These two independent Malay translations were submitted to the research team, whose members reviewed and compared them to identify and resolve any discrepancies between the two translators and then compiled them into a single reconciled forward-translated version.

The reconciled forward-translated version of the questionnaire was given to another two independent translators (one is a qualified optometrist, and the other is a certified English-Malay translator) for back translation, without reference to the original questionnaire. This helped the research team to determine if similar meaning can be derived from the reconciled version of the questionnaire. The back translation version was compared to the original questionnaire, and any discrepancies were reviewed and resolved by the research team to ensure conceptual equivalence. The original questionnaire used 7-point Likert scales; however, a consensus was reached by all team members to use a 5-point Likert scale for the translated questionnaire, which school-age children will more easily administer. According to previous study, the 5-point Likert scales is considered appropriate and generally preferred for paediatric studies [39]. Besides, a 5-point Likert scale simplifies the response process without significantly compromising its reliability [40].

### Face validity and content validity of the questionnaire

Face validity was conducted on ten adults who met the following inclusion criteria: they had normal vision (based on self-report), had no formal education in visual or health sciences and resided within the study location [41–42]. The VCQ-M and face validity form, consisting of 10 items were sent to the respondents by email. The respondents were asked to review the questionnaire for clarity and ambiguity by answering ‘Yes’ or ‘No’ for each of the item in the face validity form. The items assessed are the accuracy of grammar, spelling, and sentence structure, as well as the relevancy of the items and the difficulty level within the context of their intended use. Additionally, they reviewed the legibility of the printout, font size and spacing, and the adequacy of instruction, and structure. Feedback and responses were compiled and deliberated among the research team members, and changes were made based on consensus.

Five practising optometrists were recruited as a expert panel through purposive sampling to assess the content validity of the questionnaire items [43]. Table 2 shows the background of recruited optometrists. The expert panels were asked to evaluate the relevance of each item to the study objectives and the clarity of the statements, including any misinterpretation. They rated the relevance and clarity of each item using a four-point scale (1 = not relevant and unclear; 2 = slightly relevant and slightly unclear; 3 = relevant and clear but needs minor revision; 4 = very relevant and very clear). Additionally, they were asked to provide feedback for improvements. Content validity of the questionnaire was quantified by tabulating the content validity index (CVI) based on each item (I-CVI) and based on the scale (S-CVI). Fleiss’ Kappa and Inter-class Correlation Coefficient (ICC) values were calculated to determine the inter-rater reliability of VCQ-M. The comments and suggestions provided were discussed among the research teams, and decision were made on whether to modify or remove the items based on the ratings given for each item and suggestions by expert panels, especially if the inter-rater reliability is below the acceptable value.

**Table 2:** Background of expert panels.

Expert Panels	Age	Field of Practice	Years of experience	Field
1	32	Optometrist	10	Primary eye care
2	28	Optometrist	6	Primary eye care
3	28	Optometrist	6	Paediatric optometry
4	27	Optometrist	5	Paediatric optometry
5	27	Optometrist	5	Primary eye care

A questionnaire pre-test on normal adults was conducted to evaluate the questionnaire’s administration, assess the reliability of its measurements, and refine its content. Ten adult participants who are not working in the field related to eye healthcare and had not participated in the questionnaire validity stage of this study were recruited. The participants were seated in the examination room with controlled lighting conditions and read a paragraph randomly chosen from the Universiti Kebangsaan Malaysia (UKM) Malay Language Related Word Reading Text Test chart (Level 2) (validated by Omar et al. [44]). This study used the Xiaomi LED Smart Bulb Essential (Xiaomi Corp., Beijing, China), a smart light-emitting diode (LED) bulb with adjustable brightness up to 950 lumens. The smart LED bulb installed on a light stand were adjusted to illuminate the reading chart at 125 lux, based on measurements taken at five points (four corners and the centre). An illumination level of 125 lux was selected to accommodate individuals with visual impairment. This population may be sensitive to glare under bright lighting conditions and often requires lower illumination levels, such as 100 lux to optimise contrast sensitivity [45]. Then, a self-administered VCQ-M was given to them after five minutes of light adaptation and vision measurement. The five-minute adaptation period was chosen based on prior studies, where Stockman et al. (2007) investigated human cone light adaptation using a three-minute period [46], while Na and Suk (2014), focusing on reading performance and visual comfort, implemented a five-minute light adaptation [47]. These findings suggest a five-minute light adaptation period is appropriate for visual tasks. Subsequently, the initial internal consistency of the questionnaire was calculated based on data collected from adult participants in the pre-test to identify items that reduce the overall reliability.

**Fig 1:**
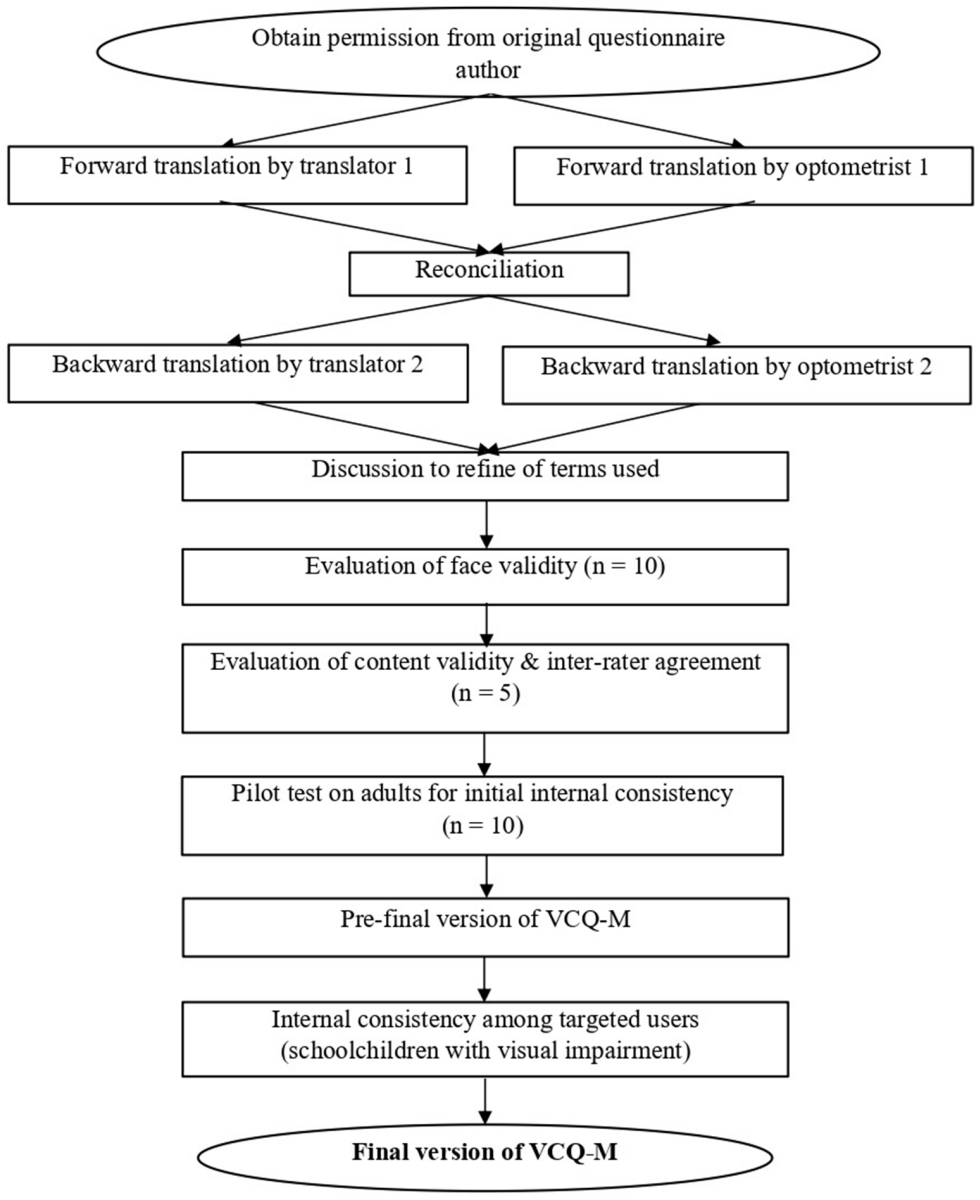
Flowchart of translation, adaptation and validation of VCQ-M.

To determine the reliability of VCQ-M among the target users, 42 subjects with visual impairment from a special secondary school in Kuala Lumpur, Malaysia, were recruited. The mean age of the participants was 14.90 ± 1.27, ranging from 13 to 18 years old. Four subjects (9.5%) had mild visual impairment, 30 (71.4%) had moderate visual impairment, and eight (19.0%) had severe visual impairment. Similar protocols to those used in the questionnaire pre-test on normal adults were performed to collect the questionnaire responses from the schoolchildren with visual impairment. Fig 1 depicts the translation, adaptation and validation process of the Visual Comfort Questionnaire into Malay.

This study focused on assessing internal consistency as part of a first-stage validation. Construct validity testing was not conducted in this phase. Therefore, the psychometric assessment of VCQ-M is partial and preliminary.

### Statistical analysis

Descriptive analysis was used to describe the demographic characteristics of subjects and determine the face and content validity of VCQ-M. IBM^®^ SPSS^®^ Statistics version 26.0 (IBM Corp., Armonk, N.Y., USA) was used to calculate Fleiss’ Kappa and Inter-class Correlation Coefficient (ICC) for inter-rater reliability, and Cronbach’s alpha of internal consistency. *P*-values of < 0.05 were considered statistically significant. In addition, ADANCO version 2.4 (University of Twente, Enschede, The Netherlands) was used to conduct composite reliability tests (Dijkstra-Henseler’s rho and Jöreskog’s rho) to confirm the internal consistency reliability of VCQ-M. Composite reliability tests provide robust cross-checking for questionnaire reliability, because Dijkstra-Henseler’s rho and Jöreskog’s rho do not assume equal item loadings and adjust for measurement error based on item-specific error variances, which is more consistent and less biased [48].

Face validity of the questionnaire was calculated by dividing the percentage of agreed item (answered ‘Yes’) over the total number of respondents. A face validity value of less than 80% (for each item or overall questionnaire) indicates poor strength of agreement, which requires restructuring of the item or the questionnaire. The strength of the agreement between 80% and 90% is substantial, and revision of the item or questionnaire is recommended, while a strength of agreement of 90% and above indicates full agreement and the items or questionnaire should be retained [49].

Content validity of the items in VCQ-M was confirmed by CVI measurement. The item-level content validity index (I-CVI) was calculated by dividing the number of agreement (scored three or four) on the item over the total number of expert panels. The scale-level content validity index (S-CVI) was calculated based on the average (S-CVI/Ave) and universal agreement (S-CVI/UA). The S-CVI/Ave value was calculated by dividing the sum of the I-CVI values for all items over the total number of items in the questionnaire, while the S-CVI/UA was calculated by dividing the number of items agreed upon by all expert panels (scored three or four) over the total number of items in the questionnaire [47]. A CVI value of 0.80 or above is accepted and indicates a valid item or scale [50].

Fleiss’ Kappa, also known as modified Kappa, was conducted to determine the inter-rater reliability of the questionnaire by assessing the agreement over what would be expected by chance. The Kappa index interpretations include poor agreement, with a Kappa index of less than 0.40, followed by good agreement, with a Kappa index between 0.40 and 0.75, while a Kappa index of more than 0.75 indicates excellent agreement [51].

An Inter-class Correlation Coefficient (ICC) was used to determine the inter-rater agreement of VCQ-M by assessing the conformity of measurements made by multiple raters. A two-way mixed model ICC with absolute agreement was selected to measure the inter-rater agreement among the panels. The ICC values interpretation includes poor reliability with ICC values less than 0.50, moderate reliability with values between 0.50 and < 0.75, good reliability with values between 0.75 and < 0.90, and values 0.90 and above indicate excellent reliability [52].

Cronbach’s alpha was initially calculated to assess the internal consistency of VCQ-M at 125 lux using data from the pre-test group of adults with normal vision. The VCQ-M was then administered to the target subjects (schoolchildren with visual impairment) to evaluate internal consistency further using Cronbach’s alpha. Dijkstra-Henseler’s rho and Jöreskog’s rho were also calculated to confirm the composite reliability and internal consistency. A minimum value of 0.70 was considered acceptable for all three reliability measures [28,48,53].

## Results

### Translation and adaptation

During the translation process, the research team members found that the syntactic component of the original English text was simple and straightforward. Thus, most of the terms used by both a qualified translator and an optometrist in forward translation came up equivalent, and the accredited Malay translation was produced with minimal difficulty during the initial translation phase. However, three items in the original questionnaire, ‘comfortable’ in item C6, ‘pleasant’ in item M2 and ‘cosy’ in item M6, had similar translations in Malay, which created a challenge during the discussion to precisely define these words in Malay. However, all items were retained for face validity and content validity to obtain feedback from the face validity participants and panels before making any decision to modify or remove the items (Table 3).

**Table 3:** Summary of items retained, revised, and eliminated in the Malay version visual comfort questionnaire.

	Item	I-CVI		Feedback	Decision
		Relevancy	Comprehensibility		
C1	I am satisfied with the light colour.	0.80	1		Retained
C2	I feel no visual distraction under current lighting level.	1	1	Similar to C5	Revised
C3	I see object visually clear under current lighting level.	1	1	Modification to the wording needed to enhance clarity.	Revised
C4	I did not experience eye fatigue under current lighting level.	1	0.80		Retained
C5	Current lighting level did not hinder reading task.	1	1	Similar to C2	Retained
C6	I feel visually comfortable under current lighting level.	1	1		Retained
VT1	I feel easy to see letter clearly under current lighting level.	1	1		Retained
VT2	I feel visual stimulation under current lighting level.	1	1	Modification to the wording needed to enhance clarity.	Revised
VT3	I feel bright for task under current lighting level.	1	1	Modification to the wording needed to enhance clarity.	Revised
VT4	I feel anxious under current lighting level.	0.43	1	Similar to M4 & M5	Eliminated
VT5	I feel visually warm under current lighting level.	0.14	0	The question is unclear and confusing	Eliminated
VT6	I feel no glare for task under current lighting level.	1	0.80	Modification to the wording needed to enhance clarity.	Revised
M1	Current lighting level make you like the space.	1	1		Retained
M2	Current lighting level make you feel pleasant.	0.29	0.86	Similar to M6	Eliminated
M3	I feel attracted to space under current lighting level.	0.14	0.57	Similar to M6	Eliminated
M4	I feel relax under current lighting level.	0.71	1	Similar to M6	Retained
M5	I feel no fidgety under current lighting level.	0.29	0.71	Similar to M4	Eliminated
M6	I feel the room is cosy under current lighting level.	1	1		Retained
M7	I feel the room is spacious under current lighting level.	1	0.80		Retained
	**S-CVI/Ave (All 19 items)**	0.78	0.87		
	**S-CVI/UA (All 19 items)**	0.63	0.63		
	**S-CVI/Ave (14 items)**	0.97	0.97		
	**S-CVI/UA (14 items)**	0.86	0.86		

Additionally, there was a challenge in discussing and reaching a consensus on the translation of item VT5 in original questionnaire. This is because the term ‘visually warm’ lacked direct equivalents in Malay, requiring cultural appropriate rewording to preserve the original meaning. According to a study from building science, visually warm refers to visual elements that evoke a perception or sensation of warmth in a space, including colours, lighting and texture [54]. Besides, visual warmth is often associated with positive emotions, such as trust and comfort [55]. As a result, the forward translation produced a direct word-for-word rendering that failed to capture the intended meaning. To address this, the research team consulted bilingual academicians to explore terms that preserve the original meaning. Based on the input, the research team selected the term they felt was close in meaning, which was later translated as ‘visually comfortable’ during the backwards translation process. This revised version was then used to collect feedback for face and content validation.

For the remaining items, both translators came up with equivalent translations grammatically and idiomatically. Therefore, we presumed that the Malay translations of VCQ-M are unambiguous and well-defined.

### Face validity

Ten complete responses were collected, followed by the results and compilation of descriptive analysis. There was 100% agreement from the respondents on the appropriateness of grammar, correct spelling, correct sentence structure, legible printout, adequacy of instruction, and instrument structure, and they were also related to the purpose of the questionnaire. The appropriateness of font size and space had 80% agreement, 70% agreement on the difficulty level of questionnaire, while clarity and unambiguity of the item had 60% agreement. The overall agreement of VCQ-M was 91%, indicated full strength of agreement [49]. Due to high overall agreement, the research team decided to proceed to content validation to obtain suggestions from the panels without further modification of the questionnaire.

### Content validity

Five responses were received from panels for content validation. Results showed that, 13 out of 19 items from the first draft of translated VCQ-M had an item-level content validity index (I-CVI) of 0.80 and above for relevance and comprehensibility. The scale-level content validity index of the 19-item VCQ-M based on average (S-CVI/Ave) was 0.78 and 0.87 for relevance and comprehensibility, respectively, and the scale-level content validity index for both relevance and comprehensibility, based on universal agreement (S-CVI/UA) was 0.63.

The research team revised the items in the questionnaire based on the feedback and suggestions provided by the panels. After reconciliation, one item (M4) ‘I feel relax under current lighting level’ was retained despite having an I-CVI value of 0.71 for relevance to the study objective, due to its high I-CVI value (1.00) for comprehensibility and there was only a single panel noted similarity to another item (M6) ‘I feel the room is cosy under current lighting level’. On the other hand, one item (VT5) was eliminated because certain expressions lacked direct equivalents in Malay and four items (VT4, M2, M3 and M5) were eliminated due to the similar meaning of questions. According to the feedback of the panels, the Malay translations of item VT4, item M5, and item M4 have similar meaning with item M6 in the original questionnaire. Additionally, Malay translations of item M2 and item M3 have a similar meaning to item M6.

Additionally, minor revisions are made to items VT2, VT3 and VT6 to enhance clarity as recommended by the panels. Four items, including VT4, M2, M3 and M5, were eliminated due to low I-CVI values and their similarity to other items in the translated questionnaire based on the expert panels feedback. Besides, the similarities were identified between items C2 and C3, as well as between items M4 and M6. However, these items had high I-CVI values, and a single panel noted the similarity between items C2 and C3; a different panel observed similarity between items M4 and M6. Therefore, only minor revision was made in item C3 after reconciliation among the research team members. The pre-final version of VCQ-M consists of 14 items.

After item revision and elimination, the scale-level content validity index based on average (S-CVI/Ave) of relevant and comprehensibility of the 14-item VCQ-M was 0.97, and the scale-level content validity index based on universal agreement (S-CVI/UA) was 0.86 for both relevant and comprehensibility, which is acceptable [50]. Table 4 shows the CVI values and feedback from the panels.

**Table 4:** Reliability test of the Malay version visual comfort questionnaire.

Parameter	Test	Value	95% Confidence Interval (CI)	Statistics	Significance	Interpretation
**Inter-rater reliability**	Fleiss Kappa, κ	0.54	0.54, 0.55	z = 7.14	*p* < 0.001	Good
		0.44	0.43, 0.44	z = 5.74	*p* < 0.001	Good
	Inter-class Correlation Coefficient, ICC(3, 5)	0.88	0.75, 0.96	*F*(13, 52) = 9.21	*p* < 0.001	Good
		0.73	0.43, 0.90	*F*(13, 52) = 3.80	*p* < 0.001	Moderate
**Internal Consistency**	Cronbach’s alpha, α	0.92				Acceptable
	Dijkstra–Henseler’s rho, ρA	0.95				Acceptable
	Jöreskog’s rho, ρc	0.94				Acceptable

### Reliability

#### Inter-rater reliability.

Fleiss’ Kappa statistic was used to determine the inter-rater reliability of VCQ-M. After elimination of five items, the Fleiss’ Kappa statistic reported good agreement among the raters for relevance (κ = 0.54, 95% CI [0.54, 0.55], z = 7.14, *p* < 0.001) and comprehensibility (κ = 0.44, 95% CI [0.43, 0.44], z = 5.74, *p* < 0.001) of VCQ-M [45]. Besides, the ICC inter-rater reliability for relevance (ICC(3, 5) = 0.88, 95% CI (0.75, 0.96), *F*(13, 52) = 9.21, *p* < 0.001) and comprehensibility (ICC(3, 5) = 0.73, 95% CI (0.43, 0.90), *F*(13, 52) = 3.80, *p* < 0.001) of VCQ-M were acceptable [52].

#### Internal consistency.

A total of 10 responses from adults with normal vision were collected under illumination level of 125 lux. Results showed that Cronbach’s alpha reliability was α = 0.92, indicating that the measurement of questionnaire was consistent for small sample [28].

The Cronbach’s alpha reliability of VCQ-M at 125 lux, tested on schoolchildren with visual impairment, showed acceptable reliability, α = 0.94. Dijkstra–Henseler’s rho (ρA) value was 0.95, supporting composite reliability [53]. Furthermore, Jöreskog’s rho (ρc) was 0.94, indicating satisfactory reliability above the 0.70 threshold [48]. Table 4 shows the results of reliability tests.

## Discussion

This study aimed to translate, adapt, and assess the validity and reliability of a visual comfort questionnaire. The 14-item VCQ-M demonstrated acceptable face validity, content validity, inter-rater reliability, and internal consistency, indicating that it is a reliable instrument (S1 Fig.). To better understand our findings, we compared them with a previous validation study involving individuals with visual impairment [56]. According to Tantirattanakulchai et al., the Thai version Impact of Vision Impairment on Children (IVI_C) reported a high Cronbach’s alpha of 0.95 for adults with visual impairment [56]. Additionally, the development of the vision function questionnaire for Indian children with visual impairment (CHVI-VFQ) by Wadhwani et al. reported Cronbach’s alpha values of 0.83 for the younger age group (aged 5–9 years) and 0.93 for the higher age group (aged 10–15 years) of children [57]. Although these previous studies did not report Fleiss’s Kappa or ICC, their use of comparable reliability metrics supports the methodological rigor of our approach. These parallels strengthen the VCQ-M’s psychometric properties and its appropriateness for assessing visual comfort in schoolchildren with visual impairment.

The current study addresses a previously unmet need in assessing visual comfort and lighting requirements, particularly among the population with visual impairment. Given the lack of standardised recommendations for classroom illumination suitable for schoolchildren with visual impairment, particularly in Malaysia, the VCQ-M will be a valuable tool for evaluating visual comfort under different illumination levels. With the aim of adapting this questionnaire for use among individuals with visual impairment, it is crucial to explore the effect of illumination level on visual functions in individuals with visual impairment.

Visual impairment is a condition where the level of vision reduced and/ or constricted, which cannot be fully corrected and reversed by corrective glasses, contact lenses, or medical and/ or surgical treatment [58]. Various approaches have been established to assist individuals with visual impairment in their visual tasks [3]. For instance, the use of optical aids that increase the retinal image size by using telescopes and magnifiers, and non-optical aids to increase the contrast of viewing the target by using additional light, tinted glasses, and reading guide [59–60]. Illumination level often impacts visual clarity and visual comfort among individuals with visual impairment, depending on their ocular condition [10,14,15,17]. Previous studies on the effect of illumination level and tinted filters on contrast sensitivity among adults with visual impairment and older adults with various ocular diseases (pseudophakia, maculopathy and glaucoma) reported that contrast sensitivity improved significantly with increased illumination level from 100 lux to 700 lux [45]. Similarly, Henry et al. (2020) found that near visual acuity, reading speed and visual satisfaction of individuals with visual impairment improved with increased illuminance [12].

Furthermore, earlier studies also reported that the average preferred illumination level of individuals with visual impairment was higher than that of normal-sighted individuals [12,16,18,45,61]. While most individuals with visual impairment preferred a higher illumination level, previous studies reported significant variability in their findings [10,12,18,61,62]. This variability can be due to some individuals with visual impairment favouring lower illumination for improved visual clarity and comfort. A recent study conducted by Lei et al. (2024) reported improvement in visual acuity and contrast sensitivity with increased illumination (800 lux) among individuals with visual impairment [63]. Therefore, validating a visual comfort questionnaire would benefit professionals such as optometrists and occupational therapists in determining the visual comfort under different lighting conditions and the specific needs of individual with visual impairment to improve their performance in visual tasks.

Previous study highlighted the importance of age-appropriate language and content to ensure schoolchildren’s comprehension and response consistency [64]. Besides, validating and assessing the internal consistency of a questionnaire for individuals with visual impairment, it is also essential to ensure that the instrument is practical and accessible. This ensures that the questionnaire is presented in a format that facilitates accessibility, cognitive clarity, and ease of use for participants with visual impairment [65]. Although the items in the VCQ-M were not initially developed with a specific focus on visual impairment, they reflect key issues and symptoms commonly experienced by schoolchildren with visual impairment during visual tasks. These include visual fatigue, glare sensitivity, visual crowding and visual distractions, factors that can significantly affect visual comfort and reading performance, especially with inappropriate lighting conditions [66–68]. By capturing these elements, the VCQ-M provides valuable insight into the visual comfort and the challenges experienced by schoolchildren with visual impairment during learning activities. Study findings indicate a high internal consistency of VCQ-M (Cronbach’s α = 0.92, Dijkstra-Henseler’s rho (ρA) = 0.95 and Jöreskog’s rho (ρc) = 0.94). In addition to Cronbach’s alpha, Dijkstra-Henseler’s rho and Jöreskog’s rho were determined, as the latent constructs within the VCQ-M cannot be directly observed but are inferred from responses to related questionnaire items. Furthermore, a minimum sample size of 100 is recommended to ensure accurate inferences in exploratory factor analysis [69]. These measures are considered more accurate indicators of reliability than Cronbach’s alpha, which assumes tau-equivalence [48,53]. This suggests that the items are highly reliable when measuring the underlying construct. Besides, the language, content, and overall presentation of the questionnaire were cognitively accessible and appropriate for schoolchildren with visual impairment, minimizing the risk of misinterpretation or random responses. The strong reliability supports the suitability of the VCQ-M for use in future studies involving comparable populations.

The VCQ-M is a valuable tool that could be easily administered by the schoolteachers and staff of schoolchildren who are sensitive to changes in lighting because of their ocular conditions. Therefore, the information obtained from the questionnaire is helpful in guiding the teachers and schools in referring the students to eye care professionals for necessary treatment and management. Consequently, eye care professionals and practitioners can develop an effective treatment plan to prevent or address visual discomfort. Quantitative measures of visual comfort could provide insight into condition progression over time and the effectiveness of treatment at each visit.

### Limitations

Although this study followed the recommended protocols for questionnaire translation and adaptation, there are study limitations during the process. Cultural bias in translation may be unavoidable due to polysemous words. However, using forward and backwards translation by blinded translators helps to minimize its impact. Nevertheless, some terms lacked direct equivalents, which could not be preserved, requiring modifications or removal. Adapting or removing items with terms that lacked direct equivalents was necessary to ensure clarity and relevance of VCQ-M within the Malay-speaking context. However, these modifications may influence the semantic equivalence of VCQ-M compared to the original questionnaire. As a result, the generalizability of the findings may be limited when applied to other multilingual or multicultural populations [69].

This study conducted a questionnaire pre-test on normal adults instead of the target questionnaire users, which is the schoolchildren with visual impairment, as recommended by the guidelines [35,70] and widely practised by the other studies [71–73]. Psychometric properties of the translated VCQ-M were also not tested in the pre-test, as the adult participants were not the targeted users of this study. The supplementary insight gained from non-target participants might not be as focused or relevant. Their perspective might not accurately capture the preferences, needs, and challenges faced by schoolchildren with visual impairment. However, the primary objective of conducting a questionnaire pre-test was to assess procedural feasibility while evaluating the questionnaire’s clarity and usability for self-administration. Although participants in the pre-test did not represent the target population, their feedback provided valuable insights into the overall structure, wording, and flow of the adapted questionnaire. Additionally, the questionnaire pre-test facilitated a preliminary assessment of internal consistency in the VCQ-M.

Another limitation of this study was that the construct validity of VCQ-M was not assessed due to the limited number of subjects. According to the rules of thumb, 100 samples and above are fair to provide adequate statistical power for data analysis [69]. However, the number of schoolchildren with visual impairment residing in the vicinity of the study location was limited, which hindered the ability to perform a construct validity test. While the questionnaire was developed based on relevant literature and panels’ input to ensure its relevance and comprehensibility, the absence of a systematic and statistical construct validation may affect the precision and extent to which the questionnaire accurately reflects the underlying theoretical constructs. This limitation could affect the generalizability of the findings. However, the high internal consistency and psychometric properties observed in this study provide strong initial evidence of the reliability and relevance of the questionnaire.

The data collection was done among the subjects with visual impairment from the same special secondary school. This was to ensure that the subjects recruited in this study were similar in educational experiences and learning environment. However, it could also lead to selection bias as the subjects included in this study might not represent the diversity of these population. This was partly because the study was conducted at the only special school for schoolchildren with visual impairment that follows Malaysia’s national secondary school curriculum. Therefore, the results might not reflect the characteristics or experiences of the excluded individuals accurately. Additionally, limited generalizability may result from the temporal and sampling biases, because the schoolchildren were available only during school hours.

Cronbach’s alpha analysis and composite reliability showed an acceptable internal consistency among the schoolchildren with visual impairment, indicating that items in VCQ-M consistently measures the same underlying construct. In this study, contextual variability, such as environment and illumination level during data collection, was controlled in the examination room. Thus, the contextual variability is not an issue in this study.

Part of this study was carried out during the COVID-19 pandemic; thus, there were restrictions and limitations due to law enforcement during the period of data collection. Future studies should be conducted in a full scale by including a larger and more varied sample across different regions and types of educational settings. This may include mainstream schools with inclusive education programs, vocational schools, and residential schools for underprivileged children. To ensure a representative and diverse sample, strategies such as collaborating with community organisations and conducting eye screening programs in different geographical areas can be employed. This would enable the development of evidence-based recommendations for improving visual comfort in schools, thereby supporting more effective learning environments for schoolchildren with visual impairment.

Future studies should conduct comprehensive construct validity assessments using larger and more diverse samples, including confirmatory factor analysis. This will enable a robust evaluation of the questionnaire’s ability to capture the intended constructs of visual comfort. Meanwhile, the findings of the current study are an important preliminary step, providing a foundation for further validation and application of the VCQ-M in a broader research context.

## Conclusion

The 14-item VCQ-M questionnaire provides a quantitative measurement of the visual comfort among individuals with visual impairment. The questionnaire may serve as a foundation for future research exploring the relationship between visual comfort and illumination in learning environments, workplaces, or other visually demanding settings.

## Supporting information

S1 Fig Malay version Visual Comfort Questionnaire (VCQ-M). (PDF)

## References

[1] Aghajari S, Chen C-C. Optimizing classroom lighting for enhanced visual comfort and reduced energy consumption. Buildings. 2025;15(8):1233. doi: 10.3390/buildings15081233

[2] Rossi S, Kara-José N, Rocha EM, Kara-Júnior N. Influence of lighting on visual performance. Arq Bras Oftalmol. 2024;87(3):e20230257. doi: 10.5935/0004-2749.2023-0257 38716966 PMC11627233

[3] Jarrard P. Tailoring in-home lighting preferences for consumers with age-related macular degeneration: using the luxiq protocol. J Vis Impair Blind. 2022;116(1):110–3.

[4] McIntosh S, Yu M, Estabrook M, Bittner AK. New insights into visually impaired patients’ preferred reading illumination and home-based reading speed with new task-lighting. Ophthalmic Physiol Opt. 2023;43(4):640–8. doi: 10.1111/opo.13111 36806302

[5] Ali LA, Mustafa FA. The state-of-the-art knowledge, techniques, and simulation programs for quantifying human visual comfort in mosque buildings: a systematic review. Ain Shams Engineering Journal. 2023;14(9):102128. doi: 10.1016/j.asej.2023.102128

[6] Blanco Cadena JD, Poli T, Košir M, Lobaccaro G, Mainini AG, Speroni A. Current trajectories and new challenges for visual comfort assessment in building design and operation: a critical review. Applied Sciences. 2022;12(6):3018. doi: 10.3390/app12063018

[7] Shafavi NS, Zomorodian ZS, Tahsildoost M, Javadi M. Occupants visual comfort assessments: a review of field studies and lab experiments. Solar Energy. 2020;208:249–74. doi: 10.1016/j.solener.2020.07.058

[8] Evans BJW, Sawyerr H, Jessa Z, Brodrick S, Slater AI. A pilot study of lighting and low vision in older people. Light Res Technol. 2020;42(1):103–19.

[9] Segura F, Sanchez-Cano A, Lopez de la Fuente C, Fuentes-Broto L, Pinilla I. Evaluation of patient visual comfort and repeatability of refractive values in non-presbyopic healthy eyes. Int J Ophthalmol. 2015;8(5):1031–6. doi: 10.3980/j.issn.2222-3959.2015.05.32 26558222 PMC4630980

[10] Şahlı E, İdil A. A common approach to low vision: examination and rehabilitation of the patient with low vision. Turk J Ophthalmol. 2019;49(2):89–98. doi: 10.4274/tjo.galenos.2018.65928 31055894 PMC6517854

[11] Fakhari M, Vahabi V, Fayaz R. A study on the factors simultaneously affecting visual comfort in classrooms: A structural equation modeling approach. Energy and Buildings. 2021;249:111232. doi: 10.1016/j.enbuild.2021.111232

[12] Henry R, Duquette J, Wittich W. Comparison of Two Lighting Assessment Methods when Reading with Low Vision. Optom Vis Sci. 2020;97(4):257–64. doi: 10.1097/OPX.0000000000001499 32304535

[13] Tidbury LP, Czanner G, Newsham D. Fiat Lux: the effect of illuminance on acuity testing. Graefes Arch Clin Exp Ophthalmol. 2016;254(6):1091–7. doi: 10.1007/s00417-016-3329-7 27106623 PMC4884565

[14] Bezan DJ, LaRussa FP, Nishimoto JH, Sendrowski DP, Spear CH. Differential diagnosis in primary eye care. Massachusetts: Butterworth-Heinemann. 1999.

[15] Bowers AR, Meek C, Stewart N. Illumination and reading performance in age-related macular degeneration. Clin Exp Optom. 2001;84(3):139–47. doi: 10.1111/j.1444-0938.2001.tb04957.x 12366325

[16] Diep M, Davey PG. Causes and Coping with Visual Impairment and Blindness. London: IntechOpen; 2018.

[17] Yanoff M. Ophthalmic diagnosis & treatment. Third ed. Panama: JP Medical Publishers. 2014.

[18] Yang SN, Jang M, Sheedy J, Seo Y. Effects of light illumination on ocular responses and visual comfort. Vision Performance Institute Research. 2019;7:1–37.

[19] Alagappen P. Design for me too! School environment for the visually impaired students. Universiti Teknologi Malaysia, Faculty of Built Environment & Surveying; 2020.

[20] Ibrahim NLN, Mohd HS, Hayder MFA. Penggunaan panduan mudah pencahayaan siang dalam reka bentuk bangunan perpustakaan berlainan era. Jurnal Kejuruteraan. 2022; 34(5(1)): 123–134.

[21] Badri KI, Mat Sulaiman MKA, Hamzah Z, Gimat MF, Qays Oleiwi M. Kesan Penggunaan Unjuran Melintang kepada Pencahayaan Siang Bilik Kuliah di Bangunan Pentadbiran, Fakulti Kejuruteraan dan Alam Bina, Universiti Kebangsaan Malaysia. jkukm. 2022;si5(1):97–103. doi: 10.17576/jkukm-2022-si5(1)-10

[22] Ludi S. Requirements gathering and domain understanding for assistive technology to support low vision and sighted students. In: Ebert A, Humayoun S, Seyff N, Perini A, Barbosa S, editors. Usability-and accessibility-focused requirements engineering: 2012 - 2014. Cham: Springer. 2016. p. 117–32.

[23] Negiloni K, Ramani KK, Sudhir RR. Environmental factors in school classrooms: How they influence visual task demand on children. PLoS One. 2019;14(1):e0210299. doi: 10.1371/journal.pone.0210299 30629656 PMC6328081

[24] Lee J-H, Moon J, Kim S. Analysis of Occupants’ Visual Perception to Refine Indoor Lighting Environment for Office Tasks. Energies. 2014;7(7):4116–39. doi: 10.3390/en7074116

[25] Abd Rahim MH, Ibrahim MI, Ab Rahman A, Yaacob NM. Translation, Cross-Cultural Adaptation and Validation of Movement Behaviour Questionnaire into Malay Language (MBQ-M) for Measuring Movement Behaviors among Preschool Children in Kelantan, Malaysia. Healthcare (Basel). 2023;11(9):1276. doi: 10.3390/healthcare11091276 37174817 PMC10178678

[26] Hassim SR, Arifin WN, Kueh YC, Yaacob NA. Confirmatory Factor Analysis of the Malay Version of the Smartphone Addiction Scale among Medical Students in Malaysia. Int J Environ Res Public Health. 2020;17(11):3820. doi: 10.3390/ijerph17113820 32481559 PMC7312542

[27] Kunyahamu MS, Daud A, Tengku Ismail TA, Md Tahir MF. Translation, Adaptation, and Validation of the Malay Version of the Barriers to Access to Care Questionnaire for Assessing the Barriers to Seeking Mental Health Care Among the Health Workforce in the East Coast Region of Peninsular Malaysia. Cureus. 2023;15(7):e41405. doi: 10.7759/cureus.41405 37546078 PMC10402845

[28] Taherdoost H. Validity and Reliability of the Research Instrument; How to Test the Validation of a Questionnaire/Survey in a Research. SSRN Journal. 2016. doi: 10.2139/ssrn.3205040

[29] Avcı AN, Memikoğlu İ. Evaluating effectiveness of LED and OLED lights on user visual comfort and reading performance. AZ. 2021;18(2):397–411.

[30] Chiou Y-S, Saputro S, Sari DP. Visual comfort in modern university classrooms. Sustainability. 2020;12(9):3930. doi: 10.3390/su12093930

[31] Erell E, Kaftan E, Garb Y. Daylighting for visual comfort and energy conservation in offices in sunny regions. In 30th PLEA International Conference – Sustainable Habitat for Developing Societies: 2014 in Ahmedabad. 2014; 16–18.

[32] Liu B, Liu Y, Deng Q, Hu K. A study on daylighting metrics related to the subjective evaluation of daylight and visual comfort of students in China. Energy and Buildings. 2023;287:113001. doi: 10.1016/j.enbuild.2023.113001

[33] Magero CV, Nyamari J, Mutisya R. Visual comfort and discomfort in public boarding secondary schools in Nairobi city County, Kenya. East Afr j health sci. 2023;6(1):62–84. doi: 10.37284/eajhs.6.1.1123

[34] Yunitsyna A, Toska A. Evaluation of the visual comfort and daylight performance of the visual art classrooms. Journal of Daylighting. 2023;10(1):117–35. doi: 10.15627/jd.2023.9

[35] World Health Organization. 2009. Process of translation and adaptation of instruments [World Health Organization]. [cited 12 August 2023]. Available from: http://www.who.int/substance_abuse/research_tools/translation/en/

[36] Aljubair JM, Aldisi D, Bindayel IA, Aldhwayan MM, Sabico S, Alsaawi TA, et al. Translation, Cultural Adaptation, and Content Validity of the Saudi Sign Language Version of the General Nutrition Knowledge Questionnaire. Nutrients. 2024;16(16):2664. doi: 10.3390/nu16162664 39203801 PMC11357498

[37] Daud A, Mohammed Nawi A, Aizuddin AN, Yahya MF. Translation, Cross-Cultural Adaptation, and Validation of the Malay-Version of the Factors Influencing Community Willingness to Perform Cardiopulmonary Resuscitation and Use an Automated External Defibrillator Questionnaire. Int J Environ Res Public Health. 2022;19(8):4882. doi: 10.3390/ijerph19084882 35457749 PMC9028418

[38] Tan YJ, Ong SC, Goh SP, Chen G, Yong VS, Khor WW, et al. Translation, cross-cultural adaptation, and psychometric validation of the Malay version of the Assessment of Quality of Life—6 Dimensions (Malay-AQoL-6D) instrument among Malaysians living with chronic heart failure. J Patient Rep Outcomes. 2024;8(1). doi: 10.1186/s41687-024-00763-3 PMC11272755 39052204

[39] Naegeli AN, Hanlon J, Gries KS, Safikhani S, Ryden A, Patel M, et al. Literature review to characterize the empirical basis for response scale selection in pediatric populations. J Patient Rep Outcomes. 2018;2:39. doi: 10.1186/s41687-018-0051-8 30238084 PMC6127069

[40] Aybek EC, Toraman C. How many response categories are sufficient for Likert type scales? An empirical study based on the Item Response Theory. International Journal of Assessment Tools in Education. 2022;9(2):534–47. doi: 10.21449/ijate.1132931

[41] Etikan I, Musa SA, Alkassim RS. Comparison of convenience sampling and purposive sampling. Am J Theor Appl Stat. 2016;5(1):1–4.

[42] Hossan D, Dato’ Mansor Z, Jaharuddin NS. Research Population and Sampling in Quantitative Study. IJBT. 2023;13(3):209–22. doi: 10.58915/ijbt.v13i3.263

[43] Abd Rahman MH, Amirtharatnam P, Sharanjeet-Kaur S, Narayanasamy S, Mohd Rasdi HF, Catherine Bastion M-L. Development of Knowledge, Attitude and Practice Questionnaire for age-related macular degeneration patients. Int J Ophthalmol. 2023;16(4):589–600. doi: 10.18240/ijo.2023.04.13 37077492 PMC10089905

[44] Omar R, Bauri N, Knight VF, Mohammed Z. Pembangunan Ujian Teks Bacaan Perkataan Berkait Bahasa Melayu Universiti Kebangsaan Malaysia. JSKM. 2015;13(1):51–6. doi: 10.17576/jskm-2015-1301-07

[45] Abraham CH, Morny E, Aboagye-MacCarthy A, Ocansey S, Ntodie M, Sakyi-Badu G, et al. The effect of filters and varying illumination on contrast sensitivity in eyes with moderate to severe visual impairment. Int Ophthalmol. 2023;43(9):3329–37. doi: 10.1007/s10792-023-02738-7 37193933

[46] Stockman A, Langendörfer M, Sharpe LT. Human short-wavelength-sensitive cone light adaptation. J Vis. 2007;7(3):4. doi: 10.1167/7.3.4 17461682

[47] Na N, Suk H-J. Adaptive luminance contrast for enhancing reading performance and visual comfort on smartphone displays. Opt Eng. 2014;53(11):113102. doi: 10.1117/1.oe.53.11.113102

[48] Hair JF, Hult GTM, Ringle CM, Sarstedt M. A primer on partial least squares structural equation modeling (PLS-SEM). Third ed. Thousand Oaks, CA: SAGE Publications. 2022.

[49] Patel N, Desai S. ABC of face validity for questionnaire. Int J Pharm Sci Rev Res. 2020;65:164–8.

[50] Yusoff MSB. ABC of Content Validation and Content Validity Index Calculation. EIMJ. 2019;11(2):49–54. doi: 10.21315/eimj2019.11.2.6

[51] Shafie S, Abd Majid F, Damio SM, Hoon TS. Evaluation on The Face and Content Validity of a Soft Skills Transfer of Training Instrument. IJARBSS. 2020;10(10). doi: 10.6007/ijarbss/v10-i10/8267

[52] Koo TK, Li MY. A Guideline of Selecting and Reporting Intraclass Correlation Coefficients for Reliability Research. J Chiropr Med. 2016;15(2):155–63. doi: 10.1016/j.jcm.2016.02.012 27330520 PMC4913118

[53] Dijkstra TK, Henseler J. Consistent Partial Least Squares Path Modeling1. MIS Quarterly. 2015;39(2):297–316. doi: 10.25300/misq/2015/39.2.02

[54] Rakotoarivelo T, Malet-Damour B. Exploring the Interplay between Thermal and Visual Perception: A Critical Review of Studies from 1926 to 2022. Buildings. 2023;13(4):879. doi: 10.3390/buildings13040879

[55] Fenko A, Schifferstein HNJ, Hekkert P. Looking hot or feeling hot: What determines the product experience of warmth?. Materials & Design. 2010;31(3):1325–31. doi: 10.1016/j.matdes.2009.09.008

[56] Tantirattanakulchai P, Hounnaklang N, Win N, Khambhiphant B, Pongsachareonnont PF. Structural validity of the impact of vision impairment questionnaire among patients with visual impairment in Thailand. Heliyon. 2024;10(16):e36353. doi: 10.1016/j.heliyon.2024.e36353 39262987 PMC11388659

[57] Wadhwani M, Vashist P, Singh SS, Gupta V, Saxena R, Kalaivani M, et al. Development of age appropriate vision function questionnaire for children with visual impairment (CHVI-VFQ). Indian J Ophthalmol. 2022;70(3):930–8. doi: 10.4103/ijo.IJO_1177_21 35225545 PMC9114577

[58] World Health Organization. International Classification of Diseases, Eleventh Revision (ICD-11) for Mortality and Morbidity Statistics. Geneva: World Health Organization. 2019.

[59] Gogate P, Shaikh M, Telap S, Gogate S, Phadke S. Low Vision and Rehabilitation. Delhi J Ophthalmol. 2022;32(5):65–71.

[60] Kaur K, Gurnani B. Low Vision Aids. Florida: StatPearls Publishing. 2022. 36256771

[61] Wittich W, St Amour L, Jarry J, Seiple W. Test-retest Variability of a Standardized Low Vision Lighting Assessment. Optom Vis Sci. 2018;95(9):852–8. doi: 10.1097/OPX.0000000000001275 30153238 PMC6133227

[62] Perlmutter MS, Bhorade A, Gordon M, Hollingsworth H, Engsberg JE, Carolyn Baum M. Home lighting assessment for clients with low vision. Am J Occup Ther. 2013;67(6):674–82. doi: 10.5014/ajot.2013.006692 24195901 PMC3819174

[63] Lei Q, Gage R, Kersten D, Legge GE. The effect of illumination on the visibility of steps and ramps for people with low vision. Optom Vis Sci. 2024;101(6):399–407. doi: 10.1097/OPX.0000000000002146 38990238 PMC12077553

[64] Al-Blaihed D, El-Housseiny AA, Farsi NJ, Farsi NM. Validity and reliability of the Arabic version of the child perceptions questionnaire for 8-10-year-old children. Qual Life Res. 2020;29(11):3131–41. doi: 10.1007/s11136-020-02545-y 32524347 PMC7591424

[65] Almpanidou S, Almaliotis D, Karamitopoulos L, Topouzis F, Konstas A-G, Labiris G, et al. Development and Validation of the Life for Low Vision Questionnaire (LIFE4LVQ) Using Rasch Analysis: A Questionnaire Evaluating Ability and Independence. J Clin Med. 2023;12(7):2549. doi: 10.3390/jcm12072549 37048633 PMC10095134

[66] Negiloni K, Ramani KK, Jeevitha R, Kalva J, Sudhir RR. Are children with low vision adapted to the visual environment in classrooms of mainstream schools?. Indian J Ophthalmol. 2018;66(2):285–9. doi: 10.4103/ijo.IJO_772_17 29380777 PMC5819114

[67] Rodrigues PFS, Pandeirada JNS. When visual stimulation of the surrounding environment affects children’s cognitive performance. J Exp Child Psychol. 2018;176:140–9. doi: 10.1016/j.jecp.2018.07.014 30149955

[68] Su B, Bao Z, Guo Y, Zheng H, Zhou J, Lu F, et al. Changes in Shape Discrimination Sensitivity Under Glare Conditions After Orthokeratology in Myopic Children: A Prospective Study. Invest Ophthalmol Vis Sci. 2023;64(1):6. doi: 10.1167/iovs.64.1.6 36626175 PMC9838587

[69] Beaton DE, Bombardier C, Guillemin F, Ferraz MB. Guidelines for the process of cross-cultural adaptation of self-report measures. Spine (Phila Pa 1976). 2000;25(24):3186–91. doi: 10.1097/00007632-200012150-00014 11124735

[70] Gaskin CJ, Happell B. On exploratory factor analysis: a review of recent evidence, an assessment of current practice, and recommendations for future use. Int J Nurs Stud. 2014;51(3):511–21. doi: 10.1016/j.ijnurstu.2013.10.005 24183474

[71] Carvalho M, Gaspar F, Potra T, Lucas P. Translation, Adaptation, and Validation of the Self-Efficacy Scale for Clinical Nurse Leaders for the Portuguese Culture. Int J Environ Res Public Health. 2022;19(14):8590. doi: 10.3390/ijerph19148590 35886442 PMC9325131

[72] Patelarou AE, Konstantinidis T, Kartsoni E, Mechili EA, Galanis P, Zografakis-Sfakianakis M, et al. Development and Validation of a Questionnaire to Measure Knowledge of and Attitude toward COVID-19 among Nursing Students in Greece. Nurs Rep. 2020;10(2):82–94. doi: 10.3390/nursrep10020012 34968353 PMC8608055

[73] Qolami M, Mirzajani A, Ronda-Pérez E, Cantó-Sancho N, Seguí-Crespo M. Translation, cross-cultural adaptation and validation of the Computer Vision Syndrome Questionnaire into Persian (CVS-Q FA©). Int Ophthalmol. 2022;42(11):3407–20. doi: 10.1007/s10792-022-02340-3 35543851 PMC9092937

