# Supplementary material for: Reliability assessment of the malay version visual comfort questionnaire for schoolchildren with visual impairment
Source: PLoS One. 2025 Oct 7;20(10):e0333228. doi: 10.1371/journal.pone.0333228 (PMC12503283; doi:10.1371/journal.pone.0333228)
Supplement: S1 Fig — (PDF) [file pone.0333228.s001.pdf]

Tahap Pencahayaan: lux

Soal Selidik Keselesaan Visual (VCQ-M)

**Nama:**	**Tarikh:**

Sila baca setiap kenyataan di bawah dan bulatkan pada nombor 1, 2, 3, 4 atau 5 bagi menggambarkan keselesaan anda di bawah pencahayaan lampu meja dan lampu carta semasa. Tiada jawapan yang betul atau salah. Jangan mengambil masa yang terlalu lama untuk menjawab mana-mana kenyataan.

Skala Pemarkahan adalah seperti berikut:

1 Sangat tidak setuju
2 Tidak setuju
3 Tidak pasti
4 Setuju
5 Sangat setuju

1	Saya berpuas hati dengan warna lampu ini.	1	2	3	4	5
2	Saya rasa penglihatan saya tidak terganggu di bawah tahap pencahayaan ini.	1	2	3	4	5
3	Saya melihat objek secara jelas di bawah tahap pencahayaan ini.	1	2	3	4	5
4	Saya tidak mengalami keletihan mata di bawah tahap pencahayaan ini.	1	2	3	4	5
5	Pencahayaan ini tidak mengganggu aktiviti membaca.	1	2	3	4	5
6	Saya rasa selesa dengan penglihatan saya di bawah tahap pencahayaan ini.	1	2	3	4	5
7	Saya rasa mudah untuk melihat huruf dengan jelas di bawah tahap pencahayaan ini.	1	2	3	4	5
8	Saya rasa tahap pencahayaan ini meningkatkan penglihatan saya.	1	2	3	4	5
9	Saya rasa tahap pencahayaan ini adalah terang untuk menjalankan tugas.	1	2	3	4	5
10	Saya rasa tidak silau untuk menjalankan tugas di bawah tahap pencahayaan ini.	1	2	3	4	5
11	Saya suka bilik/ruang ini di bawah tahap pencahayaan ini.	1	2	3	4	5
12	Saya rasa relaks di bawah tahap pencahayaan ini.	1	2	3	4	5
13	Saya rasa bilik/ruang ini selesa di bawah tahap pencahayaan ini.	1	2	3	4	5
14	Saya rasa bilik/ruang ini kelihatan luas di bawah tahap pencahayaan ini.	1	2	3	4	5
